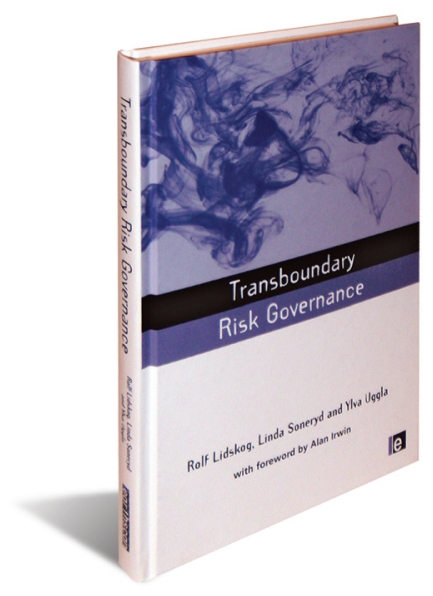# Transboundary Risk Governance

**Published:** 2010-12

**Authors:** Kristie L. Ebi

**Affiliations:** *Kristie L. Ebi, of the Carnegie Institution for Science, has been conducting research on the impacts of and adaptation to climate change for 15 years. She was a lead author for the “Human Health” chapter of the* IPCC Fourth Assessment Report*. She has edited four books on climate change and has more than 100 publications.*

The authors of this interesting, insightful, and very well-written book explore the processes of regulation of transboundary environmental risks through four case studies that engage a variety of state and nonstate actors with divergent demands and knowledge claims. The book is organized into seven chapters, with two introductory chapters on regulating risk and on making rules and generating knowledge. The case studies focus on transboundary regulation of four issues in Sweden: mobile telephones and radiation protection, oil transport in the Baltic Sea, climate change adaptation, and regulation of coexistence of genetically modified crops. The issues were chosen to reflect a range of institutional settings and structures, varying degrees to which scientific knowledge was pivotal to regulation, and historical trajectories. The book concludes with a chapter on co-producing frames, actors, and knowledge, with the lessons learned widely applicable outside Sweden.

The authors explore what gives rules authority, when and how knowledge becomes relevant (or irrelevant) to policy, and what happens when different actors have conflicting knowledge claims. They begin from the perspective that regulation of environmental risks is not a linear process in which scientists identify risks and generate the evidence base that policy makers then respond to by formulating regulations. Instead, rendering an issue or activity governable is a creative process during which the risk is framed and possible responses are constructed. The regulatory process determines what is worthy of protection, whom or what to protect, for what reason, and in what way. The negotiation of these issues is more challenging when the issues involved are complex, and with greater public engagement in the process.

National government and science are important actors in the regulatory process, but neither may play a pivotal role in the regulation of a particular issue. For transboundary risks, nation-states are both regulators and regulated. Social process—including translation, negotiation, and power struggles—determines which knowledge is relevant, the best approach for evaluating competing knowledge claims, and how best to translate a complex body of knowledge into an enforceable rule.

Regulation of environmental risks has traditionally been framed as a command-and-control activity of central government. This framing assumes that the nation-state plays a dominant role in regulation. Challenges to this view include the more than 200 international conventions concerning transboundary environmental issues. International conventions create multilevel governance where the state is only one of a wide range of actors at different levels. Some of the transboundary issues explored are relatively new, and it may not be clear which national agency or organization has the responsibility to take action.

A significant trend in regulation is to view its core mandate not as eradication but as risk management (e.g., eliminating mobile telephones would not be an acceptable option for reducing possible risks from low-frequency radiation). Risk regulation not only manages existing risks but also is viewed as an appropriate approach for controlling possible negative effects of potential future risks (e.g., genetically modified organisms). Conceptualizing an environmental exposure or hazard as a risk means that it is governable, that key uncertainties can be identified and managed. However, just gathering information on a hazard and how it can be managed is insufficient. To be useable, knowledge—particularly for complex issues—must make sense. Organizing knowledge into narratives creates a shared understanding and motivates action. The process of creating, controlling, using, and distributing frames and narratives, including negotiations over which actors, knowledge, and expertise are valid and relevant, is often hotly contested and debated.

Science plays a central role in determining the probability and magnitude of the environmental risk and in measuring the benefits and costs of various options to reduce that risk. However, significant uncertainties are associated with the complex environmental risks investigated, with ongoing debates on the sources of valid and relevant knowledge (e.g., climate change). Each case study illustrates different ways of handling scientific uncertainties and competing knowledge claims and approaches to making knowledge more policy relevant. Each also illustrates challenges to the view that scientific agreement on the dimensions of risk and possible responses is independent of policy. During regulation processes, regulatory entities are identified and empowered, narrative frames constructed, actors and their identities shaped, and expertise negotiated and established.

The authors provide concise summaries of the framing of an issue by different actors and their negotiating strategies over the character, causes, and responses for that issue. They point out that in all case studies, regulatory discussions were restricted to downstream risks (thus excluding concerns over technical, economic, and scientific development) and questions about the purpose and need for certain activities at an early stage of the process. Nevertheless, the case studies provide fascinating support for the authors’ conclusion that “Regulation is not a direct response to a predefined problem, but rather a range of actors contributing in various ways to the creation of the regulatory object.” Insights from this book will increase the effectiveness of all actors as they engage in efforts to regulate transboundary environmental risks.

## Figures and Tables

**Figure:**